# Effectiveness of influenza vaccine in preventing medically-attended influenza virus infection in primary care, Israel, influenza seasons 2014/15 and 2015/16

**DOI:** 10.2807/1560-7917.ES.2018.23.7.17-00026

**Published:** 2018-02-15

**Authors:** Hamutal Yaron-Yakoby, Hanna Sefty, Rakefet Pando, Rita Dichtiar, Mark A Katz, Yaniv Stein, Michal Mandelboim, Ella Mendelson, Tamy Shohat, Aharona Glatman-Freedman, the Israeli Influenza Surveillance Network (IISN)

**Affiliations:** 1 Department of Epidemiology and Preventive Medicine, School of Public Health, Sackler Faculty of Medicine Tel Aviv University, Tel Aviv, Israel; 2 Israel Center for Disease Control, Israel Ministry of Health, Tel Hashomer, Ramat Gan, Israel; 3 Central Virology Laboratory, Israel Ministry of Health, Chaim Sheba Medical Center, Ramat Gan, Israel; 4 Ben Gurion University, Beer Sheva, Israel; 5 School of Public Health, University of Michigan, Ann Arbor, Michigan, United States; 6 Departments of Pediatrics and Family and Community Medicine, New York Medical College, Valhalla, New York, United States; 7 The members of the group are listed at the end of the paper

**Keywords:** influenza, influenza-like illness, ILI, vaccines, immunisations

## Abstract

Influenza vaccine is recommended for the entire population in Israel. We assessed influenza vaccine effectiveness (VE) for the 2014/15 and 2015/16 seasons in Israel, for the first time. **Methods:** Combined nose and throat swab specimens were collected from patients with influenza-like illness (ILI) presenting to sentinel primary care clinics and tested for influenza virus by RT-PCR. VE of the trivalent inactivated vaccine (TIV) was assessed using test-negative case–control design. **Results:** During the 2014/15 season 1,142 samples were collected; 327 (28.6%) were positive for influenza, 83.8% A(H3N2), 5.8% A(H1N1)pdm09, 9.2% B and 1.2% A un-subtyped. Adjusted VE against all influenza viruses for this influenza season was −4.8% (95% confidence interval (CI): −54.8 to 29.0) and against influenza A(H3N2), it was −15.8% (95% CI: −72.8 to 22.4). For the 2015/16 season, 1,919 samples were collected; 853 (44.4%) were positive for influenza, 43.5% A(H1N1)pdm09, 57% B, 0.7% A(H3N2) and 11 samples positive for both A(H1N1)pdm09 and B. Adjusted VE against all influenza viruses for this influenza season was 8.8% (95% CI: −25.1 to 33.5), against influenza A(H1N1)pdm09, it was 32.3% (95% CI: (−4.3 to 56.1) and against influenza B, it was −2.2% (95% CI: (−47.0 to 29.0). **Conclusions:** Using samples from patients with ILI visiting sentinel clinics in Israel, we demonstrated the feasibility of influenza VE estimation in Israel.

## Introduction

Influenza virus infection causes morbidity and mortality worldwide every year [1]. The most effective measure of preventing influenza is the influenza vaccine. However, influenza vaccine effectiveness (VE) can vary considerably, both by season [2], and geographic location [2,3].

Israel is a country of 8.5 million people, with a Mediterranean and arid climate, located in the westernmost part of Asia. In Israel, influenza activity is seasonal, usually occurring from December through March [4] in patterns similar to those of Europe and those in neighbouring Jordan [5] and Egypt [6]. The outpatient sentinel influenza surveillance system was established in the 1996/97 season and has been conducted through primary care clinics throughout Israel. The sentinel clinics are located in all seven districts of Israel and are staffed by paediatricians, internists and family physicians. During the 2014/15 and 2015/16 influenza seasons, 23 and 26 sentinel clinics, respectively, participated in influenza surveillance. Israel's Ministry of Health recommends influenza vaccination for the entire population over the age of 6 months [7]. The trivalent inactivated vaccine (TIV), the quadrivalent inactivated vaccine (QIV) and live attenuated influenza vaccine (LAIV) are all registered for use in Israel. The inactivated vaccines against seasonal influenza are offered free of charge to all residents through clinics of the four national ‘sick fund’ organisations which are widely spread throughout the country and are similar to health maintenance organisations in the United States (US). TIV is the most widely used influenza vaccine in Israel.

In this study we evaluated, for the first time, VE against medically attended laboratory-confirmed influenza-like illness (ILI) in the community in Israel using our sentinel surveillance system. The predominant influenza strain in the 2014/15 season was a drifted influenza A(H3N2). Influenza A(H1N1)pdm2009 and influenza B co-circulated in Israel during the 2015/16 season. The overall influenza vaccination coverage in Israel during the 2014/15 and 2015/16 seasons was ca 21%. The vaccine coverage was ca 65%, 25% and 40% among individuals age 65 years of age and over, infants and children 6 months to 5 years, and individuals less than 65 years of age with chronic medical conditions, respectively [8,9].

We used the test-negative case–control design, a commonly used method for estimating influenza VE in studies that utilise sentinel surveillance systems [10].

## Methods

### Study period and population

Influenza surveillance periods lasted in Israel from 28 September 2014 until 18 April 2015, and from 27 September 2015 until 16 April 2016. During these periods, sentinel clinic providers obtained combined nasal and throat swabs [11] from a convenience sample of patients meeting the ILI case definition. The kits of combined nasal and throat swabs were provided to all sentinel clinics by the Israel Center for Disease Control. ILI was defined as a temperature of 37.8˚C and over, accompanied by one or more of the following symptoms: coryza, sore throat, cough, and muscle ache [12]. Discretion was given to physicians to include other signs or symptoms considered relevant. Sentinel clinics were asked to send up to ten samples per week.

For each patient a questionnaire with demographic, epidemiologic and clinical data was completed by sentinel medical staff. The data included date of birth, sex, date of disease onset, date of sample collection and influenza vaccine status for the evaluated season, including the date of vaccination and the type of vaccine used. For children less than 9 years of age, providers recorded whether a second dose was needed (for those receiving the influenza vaccine for the first time) and the date of the second dose, if indicated. Information regarding chronic medical conditions placing patients at risk for influenza-related complications was available for the 2015/16 season.

### Molecular identification of influenza viruses in samples obtained from sentinel patients with influenza-like illness

The combined nasal and throat samples from sentinel ILI patients were kept at 4˚C in the upright position until transport. Samples were transported once a week in cooling containers, by car, to the Central Virology Laboratory of the Israel Ministry of Health. The samples were tested for influenza by real-time RT-PCR. The viral genome was extracted from the samples during the 2014/15 season using NucliSENS easyMAG (BioMerieux, Marcy l'Etoile, France) and during the 2015/16 season using the KingFisher Purification System (Thermo Fisher Scientific, Vantaa, Finland) and the NucleoMag RNA (Macherey-Nagel, Düren, Germany) RNA extraction kit. Influenza viruses were then tested by real-time RT-PCR using Applied Biosystems 7500 Real-Time PCR system (Foster City, CA, US) and the Ambion Ag-Path Master Mix (Life Technologies, US) and TaqMan Chemistry (Foster City, CA, US) [13-16].

A subset of influenza A(H3N2) circulating in Israel during the 2014/15 season, and subsets of influenza A(H1N1)pdm09 and B circulating in Israel during the 2015/16 season underwent nt sequencing of the haemagglutinin (HA) gene. Sequenced viruses were from samples collected at various stages of each influenza season. Influenza HA gene-specific primers were used to partially amplify the influenza A and influenza B HA genes, as previously described according to World Health Organization (WHO) protocols [17].

Amplified PCR products were sequenced using ABI PRISM Dye Deoxy Terminator cycle sequencing kit (Applied Biosystems, Foster City, CA, US). Reaction mixtures were then analysed using ABI 3500 DNA Genetic Analyzer (Applied Biosystems, Foster City, CA, USA). Alignment and comparison of nt sequences were carried out using the Sequencher software version 5.4 (Gencodes Corporation, Ann Arbor, MI, US). HA sequences of reference strains used for phylogenetic analysis were obtained from the EpiFlu database of the Global Initiative on Sharing All Influenza Data (platform.gisaid.org).

### Study design

VE against influenza was assessed for individuals 6 months of age and over who received the TIV, using the test-negative case–control design [18,19]. VE was derived as (1−odds ratio (OR)) × 100, expressed as a percentage. VE was estimated for influenza A and B together and for the specific influenza subtypes, for each of the two seasons. VE was not calculated for certain influenza types and subtypes if the total number of positive samples for the type or subtype was very low, not allowing at least 5 samples in each cell of the contingency table used for OR calculation. Individuals were considered vaccinated if they received the influenza vaccine 14 days or more before disease onset. For children between the ages of 6 months and 9 years receiving the influenza vaccine for the first time, only those receiving two doses, with the second dose given 14 days or more before illness, were included in the analysis. Individuals whose samples were taken more than 7 days after of onset of symptoms were excluded from analysis. Because relatively few individuals received LAIV or QIV during the two influenza seasons, we estimated VE for TIV only.

### Statistical analysis

Percentages were compared using the Mantel-Haenszel chi-squared test. OR for crude VE calculation was performed using a univariate logistic regression model with no covariates. We adjusted for age group, sex, calendar week of sample collection, days from disease onset to swab and underlying chronic medical conditions (information regarding chronic conditions was available for the 2015/16 season only) using multivariable logistic regression. Sensitivity analysis was carried out to evaluate whether there was a difference between individuals who were swabbed on days 0–1 from symptom onset and individuals who were swabbed on days 2–7 from symptom onset. Statistical analyses were carried out using SAS version 9.4 (SAS Institute, Cary, NC, US).

### Ethical consideration

Sentinel influenza surveillance in Israel, including the testing component, is conducted in accordance with the Public Health Ordinance enacted in Israel and does not require informed consent. Molecular characterisation of influenza strains isolated from patients was approved by the ethics committee at the Sheba Medical Center (1967–15-SMC), Tel Hashomer, Israel.

## Results

### Influenza season and virus circulation

#### 2014/15 influenza season

**Figure 1:**
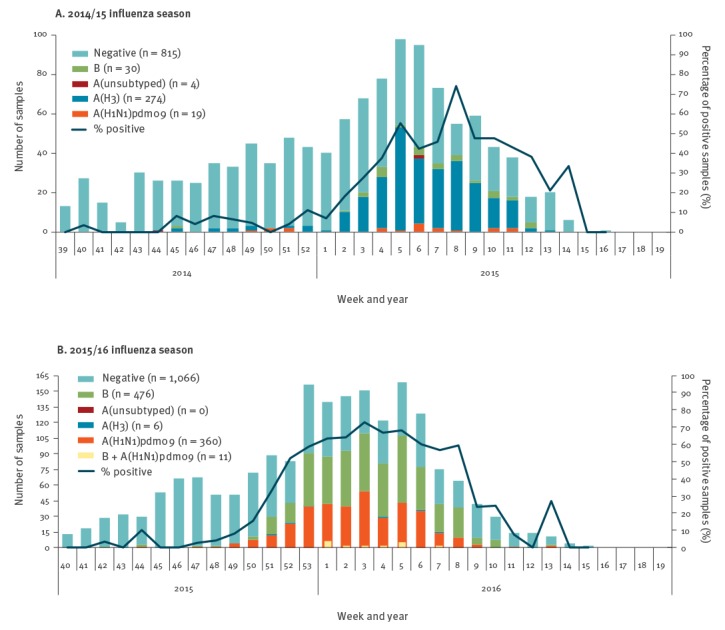
Weekly distribution of influenza-positive samples from outpatient sentinel clinics, Israel, influenza seasons 2014/15 and 2015/16

During the 2014/15 influenza surveillance season, 1,142 samples were collected from ILI patients. A total of 327 (28.6%) samples were positive for influenza, of which 297 (90.8%) samples were positive for influenza A, and 30 (9.2%) were positive for influenza B. Of the 297 influenza A samples, 274 (92.3%) were A(H3N2), 19 (6.4%) were A(H1N1)pdm09, and 4 (1.3%) were un-subtyped (Figure 1A) [20]. Characterisation of 22 influenza B samples demonstrated that 21 samples belonged to the Yamagata lineage and 1 belonged to the Victoria lineage.

Molecular characterisation of a convenience sample of 22 influenza A(H3N2) viruses from community sentinel patients, showed that all belonged to the 3C.2a clade, while the vaccine strain influenza A/Texas/50/2012(H3N2) belonged to the 3C.1 clade. A detailed description and phylogenetic tree were previously reported [21].

#### 2015/16 influenza season

Of the 1,919 samples that were collected from ILI patients during the 2015/16 influenza season, a total of 853 (44.5%) samples were positive for influenza, of which 377 (44.2%) samples were positive for influenza A, and 487 (57.1%) were positive for influenza B. Of the 377 influenza A samples, 371 (98.4%) were A(H1N1)pdm09, and 6 (1.6%) were A(H3N2) (Figure 1B) [4]. A total of 11 samples (1.3% of the positive influenza samples) were positive for both influenza A(H1N1)pdm09 and influenza B [22].

Molecular characterisation of a convenience sample of 31 influenza A(H1N1)pdm09 viruses from community sentinel patients, showed that 16 belonged to the 6B.1 clade and 15 to the 6B.2 clade, while the vaccine strain A/California/07/2009(H1N1)pdm09 belonged to clade 1. Molecular characterisation of 452 Influenza B viruses demonstrated that 394 (87.2%) belonged to the Victoria lineage, and 58 (12.8%) belonged to the Yamagata lineage, while the B components of the trivalent vaccine, B/Phuket/3073/2013, belonged to the Yamagata lineage [22].

### Study population

#### 2014/15 influenza season

**Figure 2:**
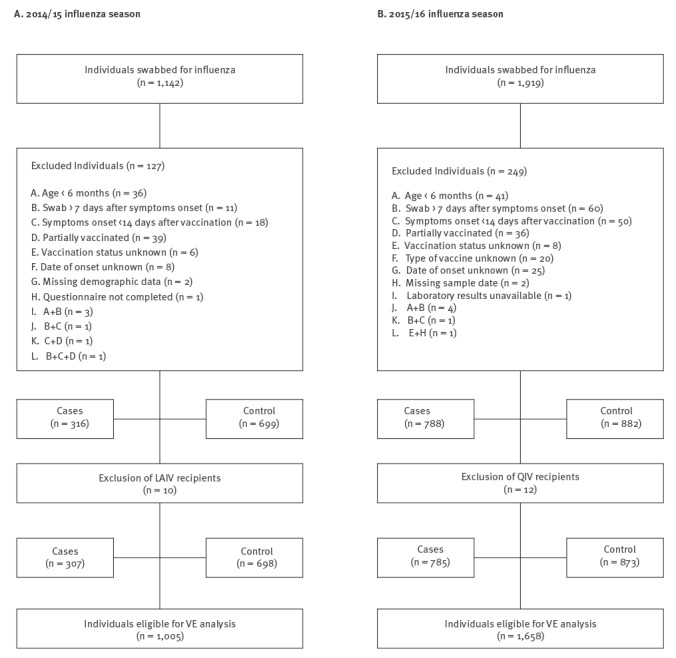
Flowchart of influenza-like illness patients from sentinel primary care clinics, Israel, influenza seasons 2014/15 and 2015/16 (n = 1,142 and 1,919, respectively)

LAIV: live attenuated influenza vaccine; QIV: quadrivalent inactivated influenza vaccine; VE: vaccine effectiveness.

Of the 1,142 samples collected from patients with ILI, 127 samples were excluded due to missing vaccination status or vaccination dates, missing day of symptom onset, sampling more than 7 days after onset of symptoms, receipt of influenza vaccine less than 14 days before disease onset, or because of partial vaccination (children less than 9 years old require two vaccine doses) (Figure 2). Of the 1,015 remaining ILI patients (316 cases and 699 controls), 10 received the LAIV (Figure 2); all 10 were less than 13.5 years of age and excluded from the study. The remaining vaccinated patients received the trivalent injected egg-grown vaccine (split or inactivated) containing an A/California/7/2009(H1N1)pdm09-like virus, an A/Texas/50/2012(H3N2)-like virus and a B/Massachusetts/2/2012-like virus. The characteristics of the 1,005 samples that were included in the assessment are presented in Table 1.

**Table 1:** Characteristics of samples from influenza-like illness patients eligible for vaccine effectiveness analysis, Israel, influenza season 2014/15 (n = 1,005)

Characteristics	Controls (n = 698)	Influenza types and subtypes of cases (n = 307)					Total (n = 1,005)	p value^c^
		Total A (n = 277)	A(H1N1)pdm09 (n = 17)	A(H3N2) (n = 257)	A(unsubtyped) (n = 3)	B (n = 30)		
	n (%)^a^	n (%)^a^	n (%)^a^	n (%)^a^	n (%)^a^	n (%)^a^	n (%)^b^	
**Age groups**								
6 months–17 years	413 (70.2)	165 (28.1)	7 (1.2)	157 (26.7)	1 (0.2)	10 (1.7)	588 (58.5)	0.83
18–44 years	193 (68.0)	76 (26.8)	7 (2.5)	68 (23.9)	1 (0.4)	15 (5.3)	284 (28.2)	
45–64 years	59 (66.3)	27 (30.3)	2 (2.2)	24 (27.0)	1 (1.1)	3 (3.4)	89 (8.9)	
≥ 65 years	33 (75.0)	9 (20.5)	1 (2.3)	8 (18.2)	0 (0)	2 (4.5)	44 (4.4)	
**Sex**								
Male	360 (69.4)	142 (27.3)	8 (1.5)	133 (25.6)	1 (0.2)	17 (3.3)	519 (51.6)	0.95
Female	338 (69.6)	135 (27.8)	9 (1.8)	124 (25.5)	2 (0.4)	13 (2.7)	486 (48.4)	
**Interval between symptom onset and swab**								
0–1 days	365 (71.0)	134 (26.1)	7 (1.4)	126 (24.5)	1 (0.2)	15 (2.9)	514 (51.1)	0.53
2–4 days	299 (67.0)	133 (29.8)	9 (2.0)	122 (27.3)	2 (0.4)	14 (3.1)	446 (44.4)	
5–7 days	34 (75.6)	10 (22.2)	1 (2.2)	9 (20)	0 (0)	1 (2.2)	45 (4.5)	
**Vaccination status^d^**								
Unvaccinated	577 (69.9)	222 (26.9)	15 (1.8)	204 (24.7)	3 (0.4)	26 (3.1)	825 (82.0)	0.47
Vaccinated (TIV)	121 (67.2)	55 (30.6)	2 (1.1)	53 (29.4)	0 (0)	4 (2.2)	180 (17.9)	

TIV: trivalent inactivated vaccine.

^a^ Percentage based on total of each row.

^b^ Percentage based on the total of 1,005.

^c^ Analysis performed for influenza positive (cases) vs negative (controls).

^d^ Individuals receiving live attenuated influenza vaccine (LAIV) (n=10) were excluded from the table and analysis.

#### 2015/16 influenza season

Of the 1,919 samples collected from ILI patients, a total of 249 samples were excluded due to missing vaccination status or vaccination dates, missing day of symptoms onset, sampling more than 7 days after onset of symptoms, receipt of influenza vaccine less than 14 days before disease onset, partial vaccination (children less than 9 years old require two vaccine doses) and missing vaccine type (Figure 2). Of the 1,670 remaining ILI patients (788 cases and 882 controls), 12 received the injectable quadrivalent egg-grown split virus vaccine (QIV) and were excluded from the study. No LAIV was available in Israel during the 2015/16 influenza season. The 242 vaccinated individuals received the trivalent injected egg-grown vaccine (split or inactivated) containing an A/California/7/2009(H1N1)pdm09-like virus, an A/Switzerland/9715293/2013(H3N2)-like virus and a B/Phuket/3073/2013-like virus. The characteristics of the 1,658 samples eligible for VE analysis are detailed in Table 2.

**Table 2:** Characteristics of samples from influenza-like illness patients eligible for vaccine effectiveness analysis, Israel, influenza season 2015/16 (n = 1,658)

Characteristics	Controls (n = 873)	Influenza types and subtypes of cases (n = 785)					Total (n = 1,658)	p value^d^
		Total A (n = 348)^a^	A(H1N1)pdm09 (n = 332)	A(H3N2) (n = 5)	A(H1N1)pdm09 + B (n = 11)	B (n = 448)^a^		
	n (%)^b^	n (%)^b^	n (%)^b^	n (%)^b^	n (%)^b^	n (%)^b^	n (%)^c^	
**Age groups**								
6 months–17 years	450 (55.2)	129 (15.8)	123 (15.1)	0 (0)	6 (0.7)	242 (29.7)	815 (49.2)	0.83
18–44 years	234 (45.0)	142 (27.3)	138 (26.5)	1 (0.2)	3 (0.6)	147 (28.3)	520 (31.4)	
45–64 years	131 (58.5)	60 (26.8)	56 (25.0)	3 (1.3)	1 (0.4)	34 (15.2)	224 (13.5)	
≥ 65 years	58 (58.6)	17 (17.2)	15 (15.1)	1 (1.0)	1 (1.0)	25 (25.2)	99 (6.0)	
**Sex**								
Male	434 (52.8)	178 (21.6)	169 (20.6)	3 (0.4)	6 (0.7)	216 (26.3)	822 (49.6)	0.91
Female	439 (52.5)	170 (20.3)	163 (19.5)	2 (0.2)	5 (0.6)	232 (27.8)	836 (50.4)	
**Chronic medical conditions**								
Yes	172 (60.6)	49 (17.2)	45 (15.8)	2 (0.7)	2 (0.7)	65 (22.9)	284 (17.1)	< 0.01
No	696 (50.9)	298 (21.8)	286 (20.9)	3 (0.2)	9 (0.7)	382 (28.0)	1367 (82.4)	
Missing	5 (71.4)	1 (14.3)	1 (14.3)	0 (0)	0 (0)	1 (14.3)	7 (0.4)	
**Interval between symptom onset and swab**								
0–1 days	386 (57.6)	132 (19.7)	124 (18.5)	0 (0)	8 (1.2)	160 (23.9)	670 (40.4)	< 0.01
2–4 days	421 (48.9)	188 (21.9)	182 (21.2)	3 (0.3)	3 (0.3)	254 (29.5)	860 (51.9)	
5–7 days	66 (51.6)	28 (21.9)	26 (20.3)	2 (1.6)	0 (0)	34 (26.6)	128 (7.7)	
**Vaccination status^e^**								
Unvaccinated	742 (52.4)	308 (21.7)	294 (20.8)	3 (0.2)	11 (0.8)	377 (26.6)	1416 (85.4)	0.62
Vaccinated (TIV)	131 (54.1)	40 (16.5)	38 (15.7)	2 (0.8)	0 (0)	71 (29.3)	242 (14.6)	

TIV: trivalent inactivated vaccine.

^a^ The 11 samples positive for influenza A(H1N1)pdm09 and influenza B are included in both the count of 'Total A' column and the 'B' column.

^b ^Percentage based on total of each row.

^c ^Percentage based on the total of 1,658.

^d^ Analysis performed for influenza positive (cases) vs negative (controls).

^e^ Individuals receiving quadrivalent inactivated influenza vaccine (QIV) (n=12) were excluded from the table and analysis.

### Vaccine effectiveness estimates against influenza virus

#### 2014/15 influenza season

For the 2014/15 season, adjusted TIV VE against influenza A and B adjusted for age group, gender and calendar week was −4.8% (95% CI: −54.8 to 29.0) (Table 3). The adjusted VE against the 2014/15 influenza A(H3N2) was −15.8% (95% CI: −72.8 to 22.4) (Table 3). We did not calculate VE for influenza A(H1N1)pdm09 or influenza B in the 2014/15 season due to the small number of positive samples (17 and 30 samples, respectively).

**Table 3:** Vaccine effectiveness estimates for trivalent inactivated vaccine based on influenza-positive and influenza-negative samples among cases and controls, Israel, influenza season 2014/15 (n =1,005)

Influenza type/subtype	Age	Cases (n =307)		Controls (n =698)		Crude VE	Adjusted VE
		Vaccinated	Unvaccinated	Vaccinated	Unvaccinated	% (95% CI)	% (95%CI)
A and B	All	59	248	121	577	−13.4 (−60.2 to 19.7)	−4.8 (−54.8 to 29.0)^a^
	6 months–17 years	24	151	68	345	19.4 (−33.4 to 51.2)	30.5 (−18.3 to 59.1)^b^
	≥18 years	35	97	53	232	−58.0 (−157.4 to 3.1)	−53.7 (−166.8 to 11.4)^b^
A(H3N2)	All	53	204	121	577	−23.9 (−77.6 to 13.6)	−15.8 (−72.8 to 22.4)^a^
	6 months–17 years	24	133	68	345	8.4 (−51.9 to 44.8)	22.7 (−31.7 to 54.6)^b^
	≥ 18 years	29	71	53	232	−78.8 (−202.3 to −5.8)	−75.7 (−216.3 to 2.4)^b^

VE: vaccine effectiveness.

^a^ Adjusted for age group, sex, calendar week and time (days) from symptom onset to swab.

^b^ Adjusted for sex, calendar week and time (days) from symptom onset to swab.

#### 2015/16 influenza season

The adjusted TIV VE against influenza A and B viruses for 2015/16 season for all ages was 8.8% (95% CI: −25.1 to 33.5) (Table 4). The adjusted TIV VE against the 2015/16 influenza A(H1N1)pdm09 was 32.3% (95% CI: −4.3 to 56.1), and the adjusted VE against the 2015/16 influenza B was −2.2% (95% CI: −47.0 to 29.0) (Table 4). Since only five individuals were infected with influenza A(H3N2), no VE analysis was performed for this subtype.

**Table 4:** Vaccine effectiveness estimates for trivalent inactivated vaccine based on influenza-positive and influenza-negative samples among cases and controls, Israel, influenza season 2015/16 (n = 1,658)

Influenza type/subtype	Age	Cases (n = 785)		Controls (n = 873)		Crude VE	Adjusted VE
		Vaccinated	Unvaccinated	Vaccinated	Unvaccinated	% (95% CI)	% (95%CI)
A and B	All	111	674	131	742	6.7 (−22.6 to 29.0)	8.8 (−25.1 to 33.5)^a^
	6 months–17 years	55	310	44	406	−63.7 (−149.9 to −7.2)	−25.0 (−98.0 to 21.0)^b^
	≥ 18 years	56	364	87	336	40.6 (14.2 to 58.8)	39.1 (7.8 to 59.8)^b^
A(H1N1)pdm09^c^	All	38	305	131	742	29.4 (−3.7 to 52.0)	32.3 (−4.3 to 56.1)^a^
	6 months–17 years	17	112	44	406	−40.1 (−154.6 to 22.9)	−8.1 (−104 to 42.7)^b^
	≥ 18 years	21	193	87	336	58.0 (30.1 to 74.7)	56.5 (24.3 to 75.0)^b^
B^c^	All	71	377	131	742	−6.7 (−46.1 to 22.1)	−2.2 (−47.0 to 29.0)^a^
	6 months–17 years	38	204	44	406	−71.9 (−173.8 to −7.9)	−25.0 (−106.8 to 24.5)^b^
	≥ 18 years	33	173	87	336	26.3 (−14.5 to 52.6)	26.5 (−20.6 to 55.2)^b^

^a^ Adjusted for age group, sex, calendar week, underlying condition and time (days) from symptom onset to swab.

^b^ Adjusted for sex, calendar week, underlying condition and time (days) from symptom onset to swab.

^c^ The 11 samples positive for influenza A(H1N1)pdm09 and influenza B in the 2015/16 season are included in the analysis of influenza A(H1N1)pdm09 and influenza B.

### Sensitivity analysis

Sensitivity analysis demonstrated substantial differences in VE estimates between individuals who were swabbed on days 0–1 days vs 2–7 days from disease onset in the 2014/15 and the 2015/16 seasons (Table 5).

**Table 5:** Sensitivity analysis of trivalent inactivated influenza vaccine effectiveness in preventing medically attended laboratory confirmed influenza, Israel, influenza seasons 2014/15 and 2015/16 (n = 1,005 and 1,658, respectively)

Influenza Season	Influenza type/subtype	Time of sample collection	Cases		Controls		Crude VE	Adjusted VE^a^
			Vaccinated	Unvaccinated	Vaccinated	Unvaccinated	% (95% CI)	% (95% CI)
2014/15	A + B	< 2 days after disease onset	34	115	59	306	−53.3 (−146.2 to 4.5)	−41.8 (−144.5 to 17.7)
		2–7 days after disease onset	25	133	62	271	17.8 (−36.6 to 50.6)	20.9 (−39.8 to 55.2)
	A(H3N2)	< 2 days after disease onset	31	95	59	306	−69.3 (−176.9 to −3.5)	−61.9 (−183.0 to 7.4)
		2–7 days after disease onset	22	109	62	271	11.8 (−50.6 to 48.3)	14.7 (−153.7 to 52.7)
2015/16	A + B	< 2 days after disease onset	32	252	52	334	18.4 (−30.5 to 49.0)	22.8 (−29.1 to 53.9)
		2–7 days after disease onset	79	422	79	408	3.3 (−35.9 to 31.2)	1.1 (−49.3 to 34.5)
	A(H1N1)pdm09^b^	< 2 days after disease onset	14	118	52	334	23.8 (−42.6 to 59.3)	−47.8 (−192.4 to 25.3)
		2–7 days after disease onset	24	187	79	408	33.7 (−8.1 to 59.3)	−41.0 (−147.4 to 19.6)
	B^b^	< 2 days after disease onset	18	142	52	334	18.6 (−44.1 to 54.0)	26.7 (−37.1 to 60.8)
		2–7 days after disease onset	53	235	79	408	−16.5 (−70.9 to 20.6)	−18.5 (−88.8 to 25.6)

^a^ Adjusted for age group, sex and calendar week for 2014/15, and adjusted for age group, sex, calendar week and underlying condition for 2015/16.

^b^ The 11 samples positive for influenza A(H1N1)pdm09 and influenza B in the 2015/16 season are included in the analysis of influenza A(H1N1)pdm09 and influenza B.

### Vaccine effectiveness estimates stratified by age

#### 2014/15 influenza season

For the 2014/15 season, the adjusted TIV VE for influenza A and B were 30.5% (95% CI: −18.3 to 59.1) for individuals less than 18 years of age, and −53.7% (95% CI: −166.8 to 11.4) for adults 18 years of age and over. The adjusted TIV VE against influenza A(H3N2) alone was 22.7% (95% CI: −31.7 to 54.6) for individuals less than 18 years of age, and −75.7% (95% CI: −216.3 to 2.4) for adults 18 years and over (Table 3).

#### 2015/16 influenza season

For the 2015/16 season, the adjusted TIV VE for individuals 18 years of age and over was 39.1% (95% CI: 7.8 to 59.8) against any influenza, 56.5% (95% CI: 24.3 to 75.0) against influenza A(H1N1)pdm09, and 26.5% (95% CI: −20.6 to 55.2) against influenza B (Table 4).

For individuals less than 18 years of age, the adjusted TIV VE was −25.0% (95% CI: −98.0 to 21.0), against any influenza, −8.1% (95% CI: −104 to 42.7) against A(H1N1)pdm09, and −25.0% (95% CI: –106.8 to 24.5) against influenza B (Table 4).

## Discussion

This was the first study in Israel to evaluate influenza VE in the community using the test-negative case–control design. In this study we evaluated two seasons with different circulation of influenza viruses. We found that in the 2014/15 season, which was characterised by the predominance of a drifted strain of influenza A(H3N2), the TIV was not effective. These findings are consistent with reported VE against community influenza A(H3N2) from the UK [18,23], Canada [24], the US [25-27], Navarra, Spain [28] and Austria [29] in the same season.

These results are also consistent with the findings of an Israeli study demonstrating that sera from Israeli individuals vaccinated with the 2014/15 injected split virus vaccine had reduced ability to neutralise the drifted influenza A(H3N2) virus [21]. Although the 2014/15 influenza vaccine was not effective against outpatient influenza A(H3N2) in our study, several studies demonstrated a better VE against preventing influenza A(H3N2)-associated hospitalisations [30,31], reaching 43% in one study [30].

For the 2015/16 influenza season, which was characterised by the co-dominance of influenza A(H1N1)pdm09 and influenza B, VE estimates varied by virus and age group. The TIV was moderately effective against influenza A(H1N1)pdm09 in adults over the age of 18 but not in those 6 months to 17 years of age. Low (and not statistically significant) TIV VE was demonstrated against influenza B in either age group.

Our TIV VE results against influenza A(H1N1)pdm09 in adults during the 2015/16 season are consistent with VE results of the inactivated vaccine against laboratory confirmed influenza A(H1N1)pdm09 in primary care setting in the US [32], Canada [33], European countries that are part of the Influenza Monitoring Vaccine Effectiveness in Europe (I-MOVE) [34] and the UK [35]. Likewise, the molecular characterisation results for influenza A(H1N1)pdm09 in Israel are consistent with other northern hemisphere results, showing that they belong to clades 6B.1 and 6B.2 [33,35,36].

Although most influenza B viruses detected in Israel during the 2015/16 season belonged to the Victoria lineage, the 2015/16 TIV contained only the Yamagata lineage. In contrast to the 2015/2016 influenza season, during the 2012/13 and 2013/14 influenza seasons, the Yamagata lineage predominated in Israel [37]. Little influenza B, mostly of the Yamagata lineage, circulated in Israel during the 2014/15 season. Thus, our low TIV VE results against influenza B may stem from a lineage mismatch between the dominant influenza B virus and the 2015/16 TIV influenza B component, along with reduced exposure to the Victoria lineage in the previous three seasons.

The low VE against influenza B in 2015/2016 in Israel differed from VE estimates from the US of 58% (95% CI: 40 to 70) against the Victoria lineage and 59% (95% CI: 45 to 69) against the Yamagata Lineage [32,38]. It also differed from VE estimate of around 50% among individuals less than 65 years of age in the UK, where, like in Israel, the Victoria lineage influenza B predominated [35]. Although, the VE of 76.5% (95% CI: 41.9 to 90.5) for those 2 to 17 years of age in the UK can be attributed, at least in part, to the use of LAIV, the VE results of individuals between the ages of 18 and 64 in the UK who mostly received the TIV [35], suggest a different mechanism of protection.

The difference in VE results against influenza A(H1N1)pdm09 between the two age groups examined in our study is interesting. The vaccine was moderately effective in adults, a finding consistent with studies from the UK and US [32,35]. However, the low VE against influenza A(H1N1)pdm09 in those less than 18 years of age in Israel differed from VE estimates in the same age group in those same countries [32,35]. Of particular note is the difference from the US study, which showed good VE among children receiving the injectable form of the influenza vaccine (while low VE estimates were found among children receiving the LAIV) [32]. It is worth noting the different vaccination rates among the adult and child controls in our study in 2015/16. While the vaccination rate among adult controls was 20.6%, it was 9.7% among control children (Table 4). By comparison, in the 2014/15 season the vaccination rates were similar in adult and children controls (18.6% and 16.5%, respectively) (Table 3). The low rate of vaccinated controls in those less than 18 years of age may have resulted in reduced precision in VE determination.

Our study has several limitations. Our sample size was initially relatively small. However, we were able to add additional sentinel clinics for the 2015/16 season and increase the sample size by ca 40%. In addition, in both seasons we had a small number of participants in certain age groups. For this reason, we estimated VE for age strata of 18 years of age and over and less than 18 years of age. Further increasing our sample size in the future will allow stratification into additional age groups, particularly for seasons in which several influenza types and subtypes co-circulate. For the 2014/15 season we did not collect data regarding chronic medical conditions. However, this information was obtained during the 2015/16 season.

We used convenience sampling to select patients presenting with ILI, similar to other studies in the field [3,18], which may have biased our sample. However, our sentinel clinics represented all seven districts in the country, and therefore, the sample likely represented larger Israeli society with respect to geography and population groups.

To evaluate whether our VE estimates might be influenced by the time elapsed from disease onset to swabbing, we conducted a sensitivity analysis. Our analysis demonstrated substantial differences in VE between patients with samples obtained 0–1 days after disease onset compared with patients with samples collected 2–7 days after disease onset. To account for these differences, we adjusted for days from disease onset to swab in our VE estimation.

We did not have information regarding influenza vaccination in previous years among ILI patients. Thus, we were unable to measure VE considering previous vaccination status. Influenza vaccination in preceding years may affect vaccine effectiveness results. Previous influenza vaccination has been associated with a negative effect on VE in several studies [24,28,39].

In conclusion, using data from a community-based influenza surveillance system in Israel, we estimated VE of inactivated influenza vaccine in the Israeli population for the first time. Our study suggests that VE may vary by season, by influenza type and subtype as well as by age. Our results, and those of others, support the need for continued efforts of estimating influenza VE, both in outpatient and inpatient settings. These efforts are necessary in order to better understand the factors that affect influenza VE and to optimise vaccine composition and use. Timely VE estimates are of paramount importance for the yearly decision regarding influenza vaccine composition.

## Notes

**Members of the Israel Influenza Surveillance Network (IISN):** Arkady Akkerman, Yoav Alkan, Shlomo Amsel, Galab Asala, Shmulik Bulvik, Lev Dynkin, Foad El-Sana, Maharan Faradian, Akiva Fradkin, Eli Gazala, Abdul Hamid Ghazawi, Michael Ginzburg, Bobi Gross, Ali Haj-Daud, Kamil Hashivon, Yael Hass, Khury Jarir, Ella Kliminski, Yoseph Laks, Tali Levenstein, Alexander Lustman, Nadia Mansour Washahi, Nir Marcus, Oded Mazor, Idit Meshulach, Kenani Nagi, Margarita Neimark, Moshe Peltz, Shiri Pergamentzev-Karpol, Nina Podbrezsky, Karen Rechavi, Eran Segal, Nirit Segal, Yuval Shilman, Eva Schlank, Raphael Singer, Paul Slater, Ran Schweid, Nitza Vadas, Oded Vagner, Ronen Yunes, Susana Zelzer, Ran Siebner.
